# Automatic and Early Detection of Parkinson’s Disease by Analyzing Acoustic Signals Using Classification Algorithms Based on Recursive Feature Elimination Method

**DOI:** 10.3390/diagnostics13111924

**Published:** 2023-05-31

**Authors:** Khaled M. Alalayah, Ebrahim Mohammed Senan, Hany F. Atlam, Ibrahim Abdulrab Ahmed, Hamzeh Salameh Ahmad Shatnawi

**Affiliations:** 1 Department of Computer Science, Faculty of Science and Arts, Najran University, Sharurah 68341, Saudi Arabia; 2 Department of Artificial Intelligence, Faculty of Computer Science and Information Technology, Alrazi University, Sana’a, Yemen; 3 Cyber Security Centre, WMG, University of Warwick, Coventry CV4 7AL, UK; hany.atlam@warwick.ac.uk; 4 Computer Department, Applied College, Najran University, Najran 66462, Saudi Arabia; iaalqubati@nu.edu.sa (I.A.A.); hsshatnawi@nu.edu.sa (H.S.A.S.)

**Keywords:** Parkinson’s disease, exploratory data analysis, coefficient of variation, t-SNE, REF, machine learning

## Abstract

Parkinson’s disease (PD) is a neurodegenerative condition generated by the dysfunction of brain cells and their 60–80% inability to produce dopamine, an organic chemical responsible for controlling a person’s movement. This condition causes PD symptoms to appear. Diagnosis involves many physical and psychological tests and specialist examinations of the patient’s nervous system, which causes several issues. The methodology method of early diagnosis of PD is based on analysing voice disorders. This method extracts a set of features from a recording of the person’s voice. Then machine-learning (ML) methods are used to analyse and diagnose the recorded voice to distinguish Parkinson’s cases from healthy ones. This paper proposes novel techniques to optimize the techniques for early diagnosis of PD by evaluating selected features and hyperparameter tuning of ML algorithms for diagnosing PD based on voice disorders. The dataset was balanced by the synthetic minority oversampling technique (SMOTE) and features were arranged according to their contribution to the target characteristic by the recursive feature elimination (RFE) algorithm. We applied two algorithms, t-distributed stochastic neighbour embedding (t-SNE) and principal component analysis (PCA), to reduce the dimensions of the dataset. Both t-SNE and PCA finally fed the resulting features into the classifiers support-vector machine (SVM), K-nearest neighbours (KNN), decision tree (DT), random forest (RF), and multilayer perception (MLP). Experimental results proved that the proposed techniques were superior to existing studies in which RF with the t-SNE algorithm yielded an accuracy of 97%, precision of 96.50%, recall of 94%, and F1-score of 95%. In addition, MLP with the PCA algorithm yielded an accuracy of 98%, precision of 97.66%, recall of 96%, and F1-score of 96.66%.

## 1. Introduction

Parkinson’s disease (PD) is a neurodegenerative disease caused by the death of neurons (called substantia nigra) that generate dopamine [1]. Dopamine is an organic chemical of the catecholamine and phenethylamine families that controls physical movement by transmitting messages between the brain and the substantia nigra, thereby enabling coordinated movement [2]. When 60–80% of the cells that produce dopamine are lost, the amount of dopamine is not enough to control a person’s movements and, thus, symptoms of PD appear [3]. The lack of dopamine neuron production leads to losing control over the body’s motor functions [4]. Four symptoms of PD are specific to the motor system, including tremors (shaking of jaws, hands, legs, and arms), rigidity (inflexibility of limbs and trunk), poor balance, and slow movement [5]. Nonmotor features of PD include dementia, depression, restless legs, temperature sensitivity, and digestion problems [6]. Although PD is still incurable, some treatment options for patients with motor and nonmotor symptoms have been developed. These options include noninvasive (drugs) and invasive (surgical) detection and treatment methods. The medications are used to block nerve impulses to control the motor system [7]. All drugs and surgical procedures have side effects. Voice disorder testing is a useful and noninvasive option for the early detection of PD since approximately 90% of PD patients have dysphonia or vocal impairment, differentiating them from healthy people [8]. Therefore, diagnosing PD through voice disorders is one of the promising and effective methods. Affecting approximately 10 million people worldwide, PD is the second type of neurodegenerative disorder after Alzheimer’s Disease [9] After age 65, people are more susceptible to the disease, and men are more susceptible than women. Common symptoms of this disease, such as loss of smell, constipation, and sleep disturbances, appear years before motor symptoms do. Thereafter, new symptoms such as tremors, imbalance, and voice impairments also appear. In the early stages, appropriate treatments that help slow the progression of the disease or stop it from developing are essential. Nevertheless, diagnosing PD according to clinical symptoms is still difficult and complex [10]. Since 90% of people with Parkinson’s have a voice disorder, detecting PD using voice data is one of the most important approaches recently [11]. In this approach, acoustic signals play an important role in the early diagnosis of PD [12]. In the early stages of PD, voice abnormalities are indistinguishable for listeners but can be discerned by analysing voice cues [13]. The PD and movement disorders are divided into two phases: the preclinical phase, where the patient suffers from neurodegeneration but there are no clinical indications, and the prodromal phase, where the patient suffers from clinical symptoms but there are not enough indications for a diagnosis [14]. Therefore, early diagnosis in both phases is essential to allow doctors to discover the disease and provide medical intervention on time. To date, there are no confirmed biomarkers to provide early detection of PD efficiently. Hence, there is an urgent need to use artificial intelligence (AI) techniques to help the healthcare sector diagnose PD early and effectively [15]. Thus, developing a computer-aided diagnostic system is necessary to analyse voice data to distinguish between PWP and healthy voices. Some researchers have recently proposed noninvasive methods for diagnosing PD using acoustic-signal analysis [16].

Although there are several efforts from researchers to provide satisfactory results for diagnosing PD, achieving better accuracy is still yet to be realized. Hence, this paper proposes novel techniques to optimize the techniques of early diagnosis of PD by differentiating patients through acoustic-data analysis to help neurologists make appropriate diagnostic decisions.

Parkinson’s disease is a neurodegenerative condition that affects a person’s movement due to a lack of dopamine production in the brain. Early diagnosis of Parkinson’s disease is crucial for timely treatment and management of the condition. However, the current diagnostic process involves multiple tests and specialist examinations, which can be time-consuming and costly. This study proposes a novel approach to early diagnosis by analyzing voice disorders associated with Parkinson’s disease. The researchers extract a set of features from recordings of a person’s voice and apply machine-learning methods to analyze and distinguish between Parkinson’s cases and healthy individuals. 

However, it is inferred to determine whether the proposed techniques, which involve feature selection, hyperparameter tuning, dataset balancing, and dimension reduction, improve the accuracy of Parkinson’s disease diagnosis based on voice analysis. Addressing a knowledge gap, the study addresses the need for improved techniques in the early detection of Parkinson’s disease. By exploring the potential of analyzing voice disorders and employing machine-learning algorithms, the study offers a novel approach to enhance diagnostic accuracy. Guiding the methodology, the purpose is to guide the selection of appropriate methods and techniques to achieve the study’s objectives. They employ feature selection, hyperparameter tuning, dataset balancing, and dimension reduction techniques to optimize the diagnostic process.

Investigating machine-learning methods that utilize feature selection and reduction techniques is of great significance in various fields, including healthcare, finance, and image recognition, among others [17]. Feature selection and reduction methods aim to identify the most relevant and informative features from a given dataset, which can improve the performance and interpretability of machine-learning models. These techniques play a crucial role in the accurate diagnosis of Parkinson’s disease [18]. One major advantage of feature selection and reduction methods is the ability to handle high-dimensional data. In many real-world applications, datasets often contain a large number of features, some of which may be redundant or irrelevant. Analyzing such datasets directly can lead to computational inefficiency, increased model complexity, and overfitting. Feature selection methods help identify a subset of features that have the most discriminatory power and contribute the most to the prediction task [19]. By reducing the dimensionality of the data, these methods can improve the computational efficiency and generalization capability of machine-learning models. Furthermore, feature selection and reduction techniques can enhance model interpretability [20]. In many domains, understanding the factors or features that contribute to a particular prediction is crucial for gaining insights into the underlying process or making informed decisions. By selecting a smaller set of relevant features, the resulting model becomes more transparent, and the relationship between the features and the target variable becomes easier to comprehend [21]. In conclusion, investigating machine-learning methods that employ feature selection and reduction techniques has significant implications across various domains. In the context of the paper on Parkinson’s disease detection, these methods can help identify the most informative acoustic features for early diagnosis. However, careful consideration of the challenges and critical issues associated with feature selection is necessary to ensure reliable and robust results.

The contributions of this paper can be summarized as follows:

- Proposing a new approach for early detection and diagnosis of PD based on acoustic signals to help doctors with early diagnosis and timely medical interventions;
- Proposing and implementing SMOTE technique for a balanced dataset;
- Apply Pearson’s coefficient to analyze the correlation between all features and remove attributes with a very high correlation;
- Apply the RFE algorithm to give each feature a percentage of its contribution to diagnosing PD;
- Apply the t-SNE and PCA algorithms to reduce the number of features in the dataset and select the features correlated with the target characteristic.

The rest of this paper is organised as follows. Section 2 provides related work. Section 3 describes the process of PD detection by dysphonia. Section 3 analyses the materials and methods applied in the study, and presents subsections on processing the features, finding correlations between them and removing the outliers. Section 4 describes the experiment setup. Section 5 presents the results of the analysis and compares the results with those of the literature. Section 6 provides the conclusion.

## 2. Related Work

This paper study is distinguished from current studies by developing diagnostic systems with various methodologies and tools that can effectively analyze audio data and distinguish between Parkinson’s and healthy people with high precision.

Hui et al. [22] proposed a CNN for analyzing the EEG recordings of 16 healthy subjects and 15 Parkinson’s patients. Gabor transform converted the EEG signals into spectral diagrams to train a CNN. Majid et al. [23] developed an approach based on spatial patterns of PD diagnosis for patients taking medication and those not taking medication. The EEG signals were processed to remove noise by a common spatial pattern. Features were extracted from the optimized signals and fed into machine-learning classifiers. The classifier achieved the best results with features extracted from beta and alpha ranges with an accuracy of 95%. Luigi et al. [24] The frequency features of the velocity and angle signals were extracted, the features were selected, and the machine-learning classifiers were optimized for FOG capture and before. The FOG detection network achieved good results and validated the patients. The network achieved a sensitivity of 84.1%, a specificity of 85.9%, and an accuracy of 86.1%. Thus, the network can predict FOG before it happens. Nalini et al. [25] utilized three deep-learning networks, RNN, LSTM, and MLP, for diagnosing the voice characteristics of PD patients. They provided loss-function curves for PD detection; the LSTM network achieved better performance than other networks. Arti et al. [26] utilized three ML and ANN algorithms to diagnose a speech dataset. Data collection and feature selection have been improved based on the wrapper and filtering method. SVM and KNN achieved an accuracy of 87.17%, while naïve Bayes had an accuracy of 74.11%. Hajer et al. [27] also used three ML algorithms for diagnosing a PD dataset to distinguish PD patients from healthy controls. The data were analyzed by linear discriminant analysis (LDA) and PCA algorithms. K-means and DBSCAN models are built based on feature-reduction algorithms. LDA performs better than PCA; thus, its output is fed into clustering algorithms. DBSCAN achieved an accuracy of 64%, a sensitivity of 78.13%, and a specificity of 38.89%. Moumita et al. [28] designed three schemes based on decision-forest and SysFor algorithms through ForestPA features for PD diagnosis. The approach requires minimal decision trees to achieve good accuracy. Increasing the density of decision trees for dynamic training and testing new samples proves to be the best method for PD detection. The decision tree with ForestPA features reached an accuracy of 94.12%. Sarkar et al. [29] collected and analysed acoustic data from 40 people: 20 with PD and 20 that were normal. The researchers used support-vector machines (SVM) and K-nearest-neighbour (KNN) classifiers to analyse and diagnose samples. To diagnose a speech disorder in PD patients, Little et al. [30] proposed an algorithm to measure dysphonia and analyse speech methods. The main objective was to distinguish between PD and normal patients through two features, namely recurrence and fractal scaling, which distinguish distorted sounds from normal sounds and applied the pitch period entropy (PPE) method to diagnose a dataset consisting of 23 patients with PD and 8 healthy subjects through the extraction of dysphonia features. The method achieved an accuracy of 91.4%. Canturk et al. [31] presented four methods for selecting features and classified the selected features into six categories. The system achieved an accuracy of 57.5% with LOSO CV and an accuracy of 68.94% with fold CV. Li et al. [32] extracted hybrid features, and then diagnosed these features through SVM; the algorithm achieved 82.5% accuracy. Benba et al. [33] extracted features by Mel-frequency cepstral coefficients and rated them by SVM, and the algorithm achieved 82.5% accuracy with LOSO CV. They also applied human factor cepstral coefficients to extract features from vowels, achieving an accuracy of 87.5% with LOSO CV. Almeida et al. [34] extracted the features of phonemic pronunciation using several methods, and PD was detected on the basis of several classifiers. Das et al. [35] applied partial least squares to reduce dimensions and applied a self-organising map (SOM) for clustering. Finally, PD was detected by the unified PD rating scale. Yuvaraj et al. [36] studied emotional information such as happiness, anger, fear, sadness, and disgust to diagnose PD and distinguish it from normal states through the use of EEG signals. Spectral decomposition was also applied with KNN and SVM classifiers, and the researchers observed that emotions were reduced in PD patients [37,38]. Yuvaraj et al. [39] applied higher-order spectra to extract features from electroencephalography signals to diagnose PD from normal cases. All classifiers achieved promising results. Sivaranjini et al. [40] used the AlexNet model to diagnose MR images to distinguish PD from normal cases; the model reached an accuracy of 88.9%. Ali et al. [41] extracted features and selected the most important ones, ranking them by the chi-square statistical method for PD diagnosis. Senturk et al. [42] applied methods to choose features and remove unessential ones, and the selected features were diagnosed through ML methods for early detection of PD. Gupta et al. [43] employed an optimised version of the crow search algorithm for early detection of PD, in which the system achieved superior accuracy.

Through previous studies, it is noted that there are limitations in the techniques used and the failure to achieve satisfactory results for the automated and early diagnosis of PD.

## 3. Proposed Approach for PD Detection

**Figure 1:**
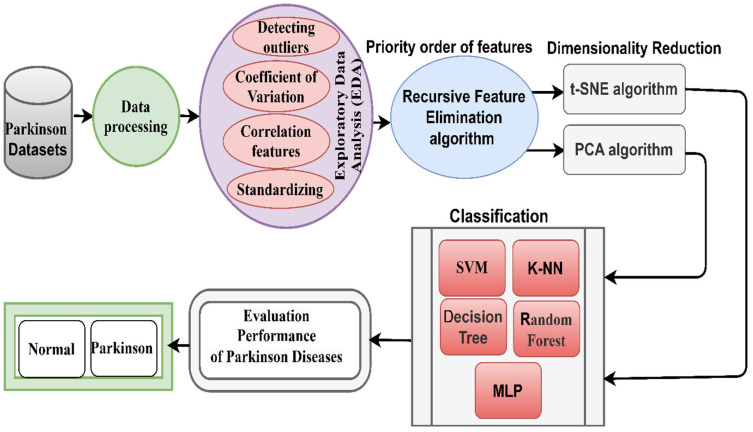
Proposed approach for early diagnosis of PD dataset.

This section, as shown in Figure 1, discusses the development of an automated technique for analyzing acoustic signals of a PD dataset for early diagnosis of PD. To improve the dataset by processing outliers and replacing missing values, a coefficient of variation was applied to measure the relative dispersion of the data points and to balance the dataset by the SMOTE method. The association of all features with the target feature was evaluated through the correlation coefficient. The relationship between features and the proportion of positive and negative correlation for each feature was measured by the RFE algorithm. To select the most critical features, t-SNE and PCA algorithms were applied. Finally, the selected features were classified by five classifiers.

### 3.1. Description of Dataset

The PD dataset used in this paper for the early detection of PD is based on voice signals, which was created and donated by Max Little of Oxford University to the UCI Machine Learning Repository [44]. The dataset is considered one of the most efficient datasets collected, prepared, and evaluated by many physicians. Many researchers have developed automated techniques and evaluated them on this dataset. It is still the destination of many researchers and those interested in the early detection of PD. The dataset of voice signals contains 195 biomedical voices which are divided into 147 phonetics for PD patients and 48 for healthy people [45]. Table 1 shows 23 features extracted from voice signals that describe the voice measure and interpretation of each feature.

### 3.2. Data Processing

Data processing is the process of converting raw data into a useful and understandable form. Data analytics is one of the most significant steps to ensure the success of subsequent measures. Data processing consists of two steps: (1) the imputation of data, which involves replacing missing values, removing outliers, and deleting duplicated values, and (2) the validation of data to ensure completeness and consistency [46]. In this paper, we noticed that the dataset does not contain duplicate values as the number of rows is similar to the number of unique column values. We also note that all the features are continuous “numerical variables” types except for the “status” feature, which is of a binary categorical type. Thus, the data of the feature must be converted to an object data type, as shown in Table 1. If discrepancies are discovered during the data-processing steps, appropriate actions are taken based on the specific nature and extent of the discrepancies. This may involve imputing missing values, handling outliers, removing duplicates, or investigating and resolving data-validation issues. The goal is to ensure the integrity and quality of the data for accurate and reliable analysis.

### 3.3. Exploratory Data Analysis (EDA)

#### 3.3.1. Detecting Outliers

The PD dataset contains 23 features of voice samples. The skewness method of statistical analysis was applied to measure the symmetry of distribution in the dataset. When feature values are on the left or right side of a median, the feature values are described as skewed. The data are symmetric when the mean, median, and mode are at the same point. The data show positive skewness when the distribution of the tail to the right side is longer or fatter, which means that the mean and median are greater than the mode [47]. 

The data show negative skewness when the tail distribution to the left side is longer or fatter than the right side, which means that the mean and median are less than the mode. In this paper, we divided the dataset into seven groups, where the features of group 1, namely, MDVP: Fo (Hz), MDVP: Fhi (Hz) and MDVP: Flo (Hz), have a right skewness, which means that the mean value is greater than the median value. Furthermore, the features of group 2, namely, MDVP: Jitter (%), MDVP: Jitter (Abs), MDVP: RAP, MDVP: PPQ and Jitter: DDP, have a right skewness, which means that the mean value is greater than the median value. Similarly, the features of group 3, namely, MDVP: Shimmer, MDVP: Shimmer (dB), Shimmer: APQ3, Shimmer: APQ5, MDVP: APQ and Shimmer: DDA, have a right skewness, which means that the mean value is greater than the median value. For group 4 features, namely, NHR and HNR, the NHR features have a right skewness, which means that the mean value is greater than the median value, whereas the HNR features have a left skewness, which means that the mean value is less than the median value. For group 5 features, namely, RPDE and D2, the median and mean are close, indicating minimal skewness. For the feature of group 6, DFA, the median and mean are close to each other, indicating negligible skewness. For the Group 7 features, namely, spread1, spread2, and PPE, the mean appears to be slightly greater than the median, and, therefore, the traits have positive skewness. Table 2 describes the skewness value for each feature.

#### 3.3.2. Coefficient of Variation 

The coefficient of variation (CV) is a statistical measurement of the relative dispersion of data points in a dataset about the mean, where the variability increases as the number increases. This measure also shows the variability of the dataset with respect to the mean. The purpose of applying CV to the features of a dataset is to assess the accuracy of the technique [48]. CV is also applied when the standard deviation is proportional to the mean, which is a measure of variability. CV is more accurate than the standard deviation. When a CV is less than 1, it has a low variance, and when a CV is higher than 1, its variance is high. Equation (1) shows the mathematical formula for CV. Table 3 describes the CV values for each feature in the dataset for healthy subjects and patients with PD.

(1) $$\mathrm{CV}=\frac{\mathrm{Std}\;\mathrm{Dev}\;}{Mean}\;\;\;\;\;\;\;\;\;$$

#### 3.3.3. Balance of Dataset

The PD dataset consists of 195 records divided into two unbalanced classes, which are healthy (Class 0 with a percentage of 24.62%) and Parkinson (Class 1 with a rate of 75.38%). Therefore, the dataset is unbalanced. Thus, the diagnostic process will tend to the majority class and ignore the minority class. Therefore, balancing the dataset is necessary. If upsampling techniques are applied, the majority of classes in the dataset will lose important information. Thus, the oversampling method overcomes this challenge. Thus, samples for the minority classes are increased. 

To overcome this problem, SMOTE was proposed and implemented [49]. It works by adding new samples to minority classes during the training phase only. This method searches for samples of minority classes and discovers the nearest neighbor to each point to generate new samples [50]. The method continues until the dataset is balanced and the minority classes become equal to the majority classes. Table 4 shows the dataset before and after the use of SMOTE. It is noted that the classes of the minority classes (healthy) became almost equal to the classes of the majority (Parkinson).

#### 3.3.4. Correlation Features

Statistical methods are used for the processing and interpretation of raw data. The correlation coefficient is a statistical indicator of relationships between features or between expected and actual values, where the correlation coefficient shows the correlation of each feature with the other. The value of the correlation coefficient varies from −1 to +1, where the relationship between two features is positive when the value of one feature increases or decreases, and the value of other features increases or decreases with it. A negative relationship occurs when the value of the feature increases, and then it decreases the other or vice versa. Zero correlation is observed when one feature does not affect the other [51]. The analysis of the correlation between all the features by tuning the Pearson’s coefficient is in Equation (2), which describes the coefficient for tuning all the features.

(2) $$\rho \;\left(X,Y\right)=\frac{\mathrm{covariance}\;\left(X,Y\right)}{\mathrm{Std}\;\left(X\right).\mathrm{Std}\;\left(Y\right)}$$

The correlation coefficient is set between 0.80 and −0.80 for positive and negative correlations, respectively. Good correlations between features are beneficial. The dataset contains highly correlated features, which must be removed before the dataset can be classified. The imbalance of the dataset is the reason for the high correlation. Thus, the oversampling process was used to balance the dataset. Then, we found the correlation coefficient again between the dataset’s features. After outliers were detected, the z-score method was applied to remove outliers by normalising the dataset. After applying the z-score method, 14 rows were removed from the dataset, which ultimately consisted of 181 rows and 23 features. Equation (3) describes the z-score method.

(3) $$z-\mathrm{score}=\frac{x-Mean\;}{\mathrm{Std}\;\mathrm{Dev}}$$

**Figure 2:**
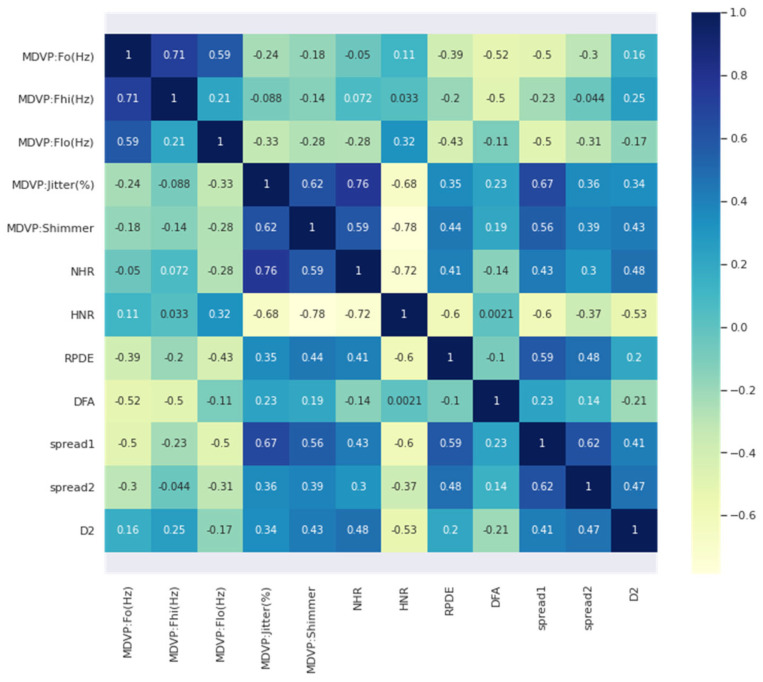
Correlation between features after removal of outliers.

After the outliers were removed, a few highly correlated features were obtained, thereby proving that the correlation coefficients were significantly affected by the outliers. Figure 2 describes a correlation between the dataset’s features after the outliers were removed. The features were reduced to 13, which then became the dataset consisting of 181 rows.

#### 3.3.5. Standardisation of Continuous Variables

Finally, the dataset obtained from the previous steps contains continuous variables; thus, the standardisation method was applied to ensure that all data had a standardised format [52]. Table 5 shows the dataset after applying the standardising method where we notice the data were formatted in the same format to give the dataset a deeper and effective meaning. The dataset was standardised by Equation (4) where each feature gets the mean subtracted from its value and divided by the standard deviation of the dataset.

(4) $$stand=\frac{\;x-\mathrm{mean}}{\mathrm{Std}\;\mathrm{Dev}\;}$$

The table provided represents a dataset related to Parkinson’s disease (PD). It appears to contain standardized continuous values for various features or variables associated with PD. Here is a breakdown of the interpretation:

- The specific values in the table represent the standardized values for each variable and observation. For example, in the first row (row 0), the standardized values for “MDVP: Fo (Hz)”, “MDVP: Fhi (Hz)”, “MDVP: Flo (Hz)”, and “MDVP: Jitter (%)” are −0.8285, −0.4355, −0.9404, and 0.8076, respectively.

The first column in the table shows the number of the data point. The next four columns show the values of four different measures of voice quality:

- MDVP: Fo (Hz) is the fundamental frequency of the voice, measured in Hertz;
- MDVP: Fhi (Hz) is the highest fundamental frequency of the voice, measured in Hertz;
- MDVP: Flo (Hz) is the lowest fundamental frequency of the voice, measured in Hertz;
- MDVP: Jitter (%) is a measure of the variability of the fundamental frequency of the voice;
- The rows are numbered from zero to four, indicating different instances or observations within the dataset;
- Each cell in the table represents a value corresponding to a specific variable and observation. The values are standardized, which means they have undergone a process of normalization or scaling to a common scale, often with a mean of zero and a standard deviation of one. Standardization is performed to facilitate comparisons and analysis of variables with different scales or units.

Without further context or information about the dataset, providing a more detailed interpretation or analysis is challenging. However, based on the given information, it can be inferred that this dataset contains standardized continuous values related to PD and its associated variables, potentially for further analysis, modelling, or statistical calculations.

The table shows that the values of all eight measures of voice quality are different for each data point. This suggests that there is a wide range of voice quality in the PD dataset. The standardization process has helped to make the values in the table more comparable, which will make it easier to analyze the data.

#### 3.3.6. Recursive-Feature Elimination Algorithm

After the preprocessing, it is necessary to identify the correlation between the features and the percentage of positive and negative correlation of all features. Therefore, finding the correlation of all features with the target feature (status) is necessary to specify the contribution of each feature in diagnosing the condition of Parkinson’s. In this paper, we applied the RFE algorithm, which works to find the correlation of all features with the target feature. The RFE algorithm is easy to use, effective, and efficient to select the most important features correlated to the effective prediction of the target feature and eliminate the features that have a weak correlation with the status feature [53]. Table 6 shows the correlation of all dataset features with the target feature. It is noticed that there is a positive and negative correlation between the features and the status feature; it is worth noting that the best positive features correlated to status are spread1, MDVP: Fo(Hz), MDVP: Flo(Hz), etc. It is worth noting that we applied this algorithm to the dataset after removing the features that contain many outliers.

### 3.4. Dimensionality Reduction

#### 3.4.1. t-SNE Algorithm

Dimensional reduction algorithms select the most important features strongly associated with the target characteristic. Thus, important and highly representative features are obtained to obtain high accuracy. Dimensional reduction is referred to as reducing the number of variables in a dataset. The dataset after dimension reduction can have better predictive performance than the original dataset [54].

In this paper, the t-SNE algorithm was proposed and applied to reduce the dimensions of nonlinear data and drops the data from a high-dimensional space P to a low-dimensional data space Q to visualize the data. Through the name of the algorithm, which means that the probability is random and not confirmed, and is concerned with the variance of neighbourhood points and the inclusion of data in a low space. In addition, t-SNE generates different data each time for the same dataset but it is focused on keeping adjacent data points. The algorithm distributes the Xi and Xj pairs, assigning the similar features in a higher probability and the dissimilar features in a lower probability. Equation (5) describes the pairwise similarity in the high-dimensional data space, and X’s conditional probability has many neighbors. Equation (6) also illustrates the representation of data points in a low-dimensional space by t-SNE. Then, t-SNE iteratively operates the same probability distribution over a smaller data space to reduce the Kulback–Leibler (KL) variance, as shown in Equation (7). The algorithm reduced the features of the new dataset from 12 to 10 features.

(5) $$P\left(x_{i\;}/x_{j\;}\right)=\frac{\;S\left({\;x}_{i\;},{\;x}_{j\;}\right)}{\sum_{m\neq i}^{N}S\left({\;x}_{i\;},{\;x}_{m}\right)}$$

(6) $$Q\left(y_{i\;}/y_{j\;}\right)=\frac{\;S\left({\;y}_{i\;},{\;y}_{j\;}\right)}{\sum_{m\neq i}^{N}S\left({\;y}_{i\;},{\;y}_{m}\right)}$$

(7) $$KL=\underset{i}{\sum }\underset{j}{\sum }\;P\left({\;x}_{i\;},{\;x}_{j\;}\right)\;log\frac{\;P\left(x_{i\;}/x_{j\;}\right)}{\;Q\left({\;y}_{i\;},{\;y}_{j\;}\right)}\;\;\;\;\;\;\;$$

where $P\left(x_{i\;}/x_{j\;}\right)$ is the high-dimensional data space and $x_{i\;}/x_{j\;}$ are pairs in the P space; $Q\left(y_{i\;}/y_{j\;}\right)$ is the low-dimensional data space and $y_{i\;}/y_{j\;}\;$are pairs in the Q space.

#### 3.4.2. PCA Algorithm

One of the popular dimensionality reduction algorithms, PCA, is an unsupervised statistically working algorithm that converts the values of correlated features into linearly uncorrelated features called principal components. The algorithm depends on the mathematical concepts of variance, covariance, eigenvalues, and eigenvectors. The dimensions refer to the number of features in the dataset. The correlation refers to the correlation between two features; when components are orthogonal, the relationship between the two features is zero [55]. The algorithm standardizes the dataset so that the features are of high variance. However, if the variance is independent of the significance of the features, then the algorithm will divide each distinct value by the standard deviation of all the features. The Z covariance matrix contains the variance between two pairs of features [56]. Eigenvectors represent axes of information with a high variance that have eigenvalues. The algorithm arranges the eigenvalues in descending order and the eigenvectors in descending order in the P matrix. After that, the Z covariance matrix is multiplied by the P matrix to get new features. Finally, essential and relevant features are preserved and less critical features are removed to produce a new dataset. In this paper, the features were reduced from 12 to 9 while retaining the most vital information.

### 3.5. Classification Algorithms

The aim of classification is to find a model that describes and, at the same time, distinguishes classes of data, and then uses it to predict the class to which an unclassified object will belong. Classification is a process that allows data to be divided into given classes based on their properties [57]. The classification process takes place in the following steps:

- Training based on the analysis of the so-called classification model created in the training set;
- Testing, that is, evaluation of the quality of the created model using test data.

In this paper, five types of classification algorithms that are most widely used in the literature were applied for the early diagnosis of PD.

#### 3.5.1. Support-Vector Machines 

The SVM has a straight line in the middle called a hyperplane that separates two classes so that the margin minimum is maximised. These classes are normal and stroke. This hyperplane is a decision boundary found by the SVM algorithm. The decision boundary divides the data space into two halves called normal and infected cases. The geometric mean edge is the distance from the decision boundary to the nearest data point. When the resolution limits are separated (hyperplane) and the training data are linearly separable, the geometric edge is positive. The goal is to find a hyperplane that maximises the margin. When the training data are linearly separable, a single linear decision limit exists, which separates the normal data above the hyperplane and the data of stroke patients under the hyperplane [58].

#### 3.5.2. K-Nearest Neighbour

The KNN algorithm used a supervised ML method to solve regression and classification problems. KNN does not include any knowledge stage. This algorithm detects similarities between the new data point and the stored data points [59]. The similarities indicate how close or far the new point (test data) is from the stored data (training data) based on Euclidean distance. In other words, the closer the new data point is to any training point, the more closely it belongs to this class. 

The KNN algorithm is applied through the following steps:

- The value of the variable K is set;
- The Euclidean distance from the new data point to the training data points is calculated;
- The sorted group is organised in ascending order based on the calculated Euclidean distance;
- The K-value labels are added;
- According to the value of K, the new data point is classified into its nearest neighbours according to the Euclidean distance.

#### 3.5.3. Decision Tree 

DT is a diagram with a tree structure, where the uppermost node represents the root. Internal nodes represent feature testing, edges represent test results, and leaf nodes indicate the class into which it was classified. The key point in creating the DT is selecting the “highest decision” feature, which initially forms the root node of the tree. If we can find such a feature, then the process of determining the correct class for a given classification object becomes highly accurate and computationally less demanding [60]. The process moves from the root node according to certain rules to the leaf node where the decision is made.

#### 3.5.4. Random Forest 

RF is a generalisation of the bagging methodology, which generates many trees based on the CART algorithm and then collects predictions or ratings from each tree. The intention is to reduce the high variance generated from each individual tree, and thus improve diagnostic performance. Forests are random, similar to bootstrap as a clustering method, which is attractive mainly because they are able to strengthen weak methods, thereby resulting in accurate diagnostic predictions [61] Finally, the RF takes the vote from each tree and the diagnosis is made according to the highest vote output from all the trees in the RF. 

#### 3.5.5. Multilayer Perceptron

The MLP is a generalisation of the perceptron for solving nonlinear separable problems. The perceptron contains an input layer to receive the input data, hidden layers to address the problem, and an output layer to show the results. 

The MLP architecture consists of the following parts:

- An input layer, which is only responsible for receiving input signals, whether images or text, and passing them to the next layer;
- An output layer that provides the network response and shows the required diagnostic results;
- Hidden layers, which works on processing the entered data to find solutions to the given problem;
- Feedforward networks, which enable communication where the data move only in the forward direction; and

Neurons that are connected to all the other neurons in the next layer.

## 4. Experimental Results

The results of the system development are presented in this section.

### 4.1. Experiment Settings

The experiment was conducted on a computer running the Windows operating system with the hardware equipment that can be seen in Table 7. The experiments were coded using Python. 

### 4.2. Splitting Dataset

The dataset generated by analyzing audio signals consisted of 195 records divided into two unbalanced categories, healthy and Parkinson’s. This dataset was divided into 69.75% for training and 30.25% for testing. Before balancing, the dataset contains 147 (75.38%) PD records and 48 healthy records (24.62%). While after balancing the dataset, the dataset contains equal records during the training phase, with 103 records for both classes. Table 4 describes the distribution of dataset samples during the training and testing phases.

### 4.3. Evaluation Metrics

The performance of the classification algorithms on the PD dataset was evaluated by using four statistical measures: accuracy, precision, recall, and F1 score. These evaluation metrics are the most effective measure to test the effectiveness of classification models. Equations (8)–(11) describe the process of calculating the statistical metrics, where TP (true positive) and TN (true negative) represent correctly classified instances, whereas FP (false positive) and FN (false positive) represent incorrectly classified instances [62].

(8) $$\mathrm{Accuracy}=\frac{\mathrm{TN}\;+\;\mathrm{TP}}{\mathrm{TN}\;+\;\mathrm{TP}\;+\;\mathrm{FN}\;+\;\mathrm{FP}}\;\times 100\%\;\;\;$$

(9) $$\mathrm{Precision}=\frac{\mathrm{TP}}{\mathrm{TP}\;+\;\mathrm{FP}}\;\times 100\%$$

(10) $$\mathrm{Recall}\;=\frac{\mathrm{TP}}{\mathrm{TP}\;+\;\mathrm{FN}}\times 100\%$$

(11) $$F1\;\mathrm{score}=2\times \frac{\mathrm{Precision}\;\times \mathrm{Recall}\;}{\mathrm{Precision}\;+\;\mathrm{Recall}\;}\;\times 100$$

where TP refers to Parkinson’s instances that are correctly classified, TN indicates normal instances that are correctly classified, FN represents PD instances that are classified as normal, and FP indicates normal instances that are classified as PD.

### 4.4. Leave-One-Subject-Out Cross-Validation

The leave-one-subject-out cross-validation (LOSO CV) method is a type of cross-validation that is commonly used to evaluate the performance of machine-learning models on the acoustic signals dataset for Parkinson’s disease. In LOSO CV, the model is trained on all but one subject’s data; then, the model’s performance is evaluated on the held-out subject’s data. This process is repeated for each subject in the dataset. The final accuracy of the model is calculated as the average accuracy across all subjects. The LOSO CV method is a more robust evaluation method than other cross-validation methods, such as k-fold cross-validation, because it accounts for the variability between subjects. This is important because the acoustic signals dataset contains a wide range of subjects with different vocal characteristics. The LOSO CV method can help to ensure that the model is not overfitting to the training data and that it is able to generalize to new subjects.

Here is a step-by-step description of the LOSO CV method on the acoustic signals dataset for Parkinson’s disease:

1. Dataset preparation: the acoustic signals dataset contains recordings of acoustic features extracted from the speech signals of individuals with and without Parkinson’s disease. Each subject in the dataset contributes multiple recordings;
2. Subject split: the dataset is divided into subjects, where each subject corresponds to an individual participant. In LOSO CV, one subject is selected as the test subject, and the remaining subjects are used as the training set. This process is repeated for each subject in the dataset, ensuring that each subject serves as the test subject once;
3. Training phase: for each iteration of LOSO CV, the machine-learning model is trained on the training set, which consists of all subjects except the test subject. The model learns the patterns and relationships between the acoustic features and the presence of Parkinson’s disease;
4. Testing phase: after training the model, the test subject is used to evaluate the model’s performance. The model takes the acoustic features from the test subject’s recordings as input and predicts whether or not the subject has Parkinson’s disease;
5. Performance evaluation: the predictions made by the model are compared to the true labels (i.e., the presence or absence of Parkinson’s disease) for the test subject. Common evaluation metrics such as accuracy, precision, recall, and F1 score can be calculated to assess the model’s performance on the test subject;

Iteration: steps 2–5 are repeated for each subject in the dataset, with each subject serving as the test subject exactly once. The performance metrics obtained for each iteration can be aggregated to get an overall assessment of the model’s performance on the acoustic signals dataset. Overall, the LOSO CV method is a valuable tool for evaluating the performance of machine-learning models on the acoustic signals dataset for Parkinson’s disease. It is a more robust evaluation method than other cross-validation methods, and it can help to ensure that the model is not overfitting to the training data and that it can generalize to new subjects.

### 4.5. Results

#### 4.5.1. Results of Classifiers with the t-SNE Method

**Figure 3:**
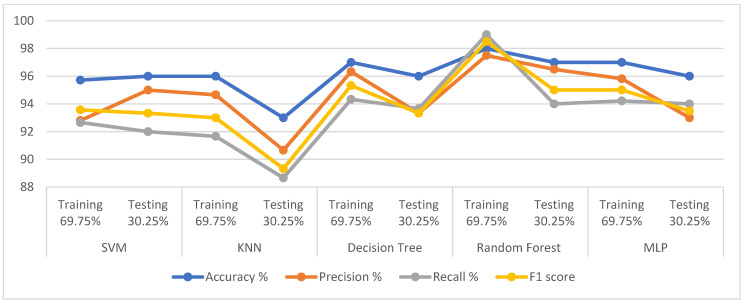
Displaying the performance of the classification algorithms on the dataset using classifiers with t-SNE.

The dataset was divided into 69.75% for training and 30.25% for testing and the SMOTE technique was applied to balance the dataset during the training phase. Each feature was arranged and given a percentage according to its relation to the target feature using the RFE algorithm. The t-SNE algorithm also reduced the dataset dimensions and the features to ten important features. The SVM, KNN, DT, RF, and MLP classifiers were fed with 10 features. The loss function was also reduced by adjusting the hyperparameter of the classifiers during the training phase. Table 8 describes the results achieved by the five mentioned classification algorithms. The RF algorithm achieved the best results during the training and testing phases than the rest. During the training phase, RF achieved accuracy, precision, recall, and F1-score of 98%, 97.50%, 99%, and 98.50%, respectively, while during the testing phase, it reached of 97%, 96.50%, 94%, and 95%. During the training phase, DT achieved accuracy, precision, recall, and F1-score of 97%, 96.33%, 94.33%, and 95.33%, respectively, while during the testing phase, it reached 96%, 93.33%, 93.66%, and 93.33%. During the training phase, MLP achieved accuracy, precision, recall, and F1-score of 97%, 95.82%, 94.22%, and 95.00%, respectively, while during the testing phase, it reached 96%, 93%, 94%, and 93.49%. During the training phase, SVM achieved accuracy, precision, recall, and F1-score of 95.72%, 92.80%, 92.66%, and 93.57%, respectively, while during the testing phase, it reached 96%, 95%, 92%, and 93.33%. Finally, KNN obtained accuracy, precision, recall, and F1-score during the training phase of 96%, 94.66%, 91.66%, and 93%, respectively, while during the testing phase, it reached 93%, 90.66%, 88.66%, and 89.33%. Figure 3 shows that all the classifiers were accurate and effective in categorising PD cases and they achieved more accurate diagnostic results than the classification of normal patients.

#### 4.5.2. Results of Classifiers with the PCA Method

**Figure 4:**
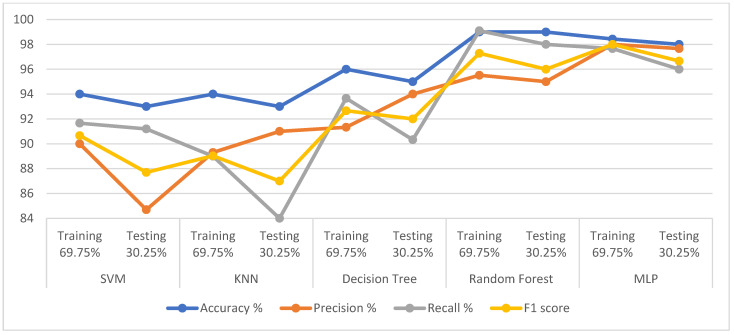
Displaying the performance of the classification algorithms on the dataset using classifiers with PCA.

In this experiment, the processing stages of the dataset passed through the same stages, except for the dimensionality-reduction process, where the high-dimensional data space was represented in the low-dimensional data space by the PCA algorithm. The PCA algorithm reduced the dataset’s features to nine important features for diagnosing PD. The loss function in classification algorithms was also reduced by adjusting the hyperparameter of the classifiers during the training phase. Table 9 describes the results achieved by the five algorithms. The RF algorithm achieved the best results during the training and testing phases than the rest. The RF and MLP algorithms achieved the best results during the training and testing phases than the rest. During the training phase, RF achieved accuracy, precision, recall, and F1-score of 99%, 95.52%, 99.10%, and 97.28%, respectively, while during the testing phase, it reached 99%, 95%, 98%, and 96%. During the training phase, MLP achieved accuracy, precision, recall, and F1-score of 98.43%, 98%, 97.66%, and 98%, respectively, while during the testing phase, it reached 98%, 97.66%, 96%, and 96.66%. During the training phase, DT achieved accuracy, precision, recall, and F1-score of 96%, 91.33%, 93.66%, and 92.66%, respectively, while during the testing phase, it reached 95%, 94%, 90.33%, and 92%. During the training phase, SVM achieved accuracy, precision, recall, and F1-score of 94%, 90%, 91.66%, and 90.66%, respectively, while during the testing phase, it reached 93%, 84.70%, 91.20%, and 87.70%. Finally, KNN obtained accuracy, precision, recall, and F1-score during the training phase of 94%, 89.30%, 89%, and 89.03%, respectively, while during the testing phase, it reached 93%, 91%, 84%, and 87%. Figure 4 shows that all classifiers achieved accurate and effective diagnostic results for diagnosing PD.

## 5. Discussion

PD is a health problem that threatens the elderly and is caused by neurodegeneration due to the death of neurons that secrete dopamine. The manual diagnosis of PD is still lacking, doctors’ opinions differ, and the number of doctors in developing countries is small. Thus, automated diagnosis by artificial intelligence solves these challenges. In this study, many systems have been developed that go through many stages of image processing. The PD dataset went through data optimization and feature correlation rents. The RFE algorithm was applied to give the percentage contribution of each feature to the target feature. The features of the dataset were subjected to the t-SNE and PCA algorithms to select the most important features and fed to five classifiers for classification. When analyzing acoustic signals for the diagnosis of Parkinson’s disease, t-SNE and PCA are both dimensionality reduction techniques that can be employed. However, they serve different purposes and have distinct advantages and limitations. Complementary information: t-SNE and PCA capture different aspects of the data. While t-SNE is effective at visualizing clusters and preserving the local structure, it may not capture the overall variance or global patterns in the data. PCA, on the other hand, focuses on explaining the maximum variance in the data, which can provide valuable insights into the most significant features. By combining both techniques, you can benefit from their complementary information. Interpretability: PCA produces orthogonal components that are interpretable as linear combinations of the original features. These components represent the directions of maximum variance in the data. In contrast, t-SNE does not provide straightforward interpretations or explicit relationships with the original features. Therefore, PCA can help in understanding the underlying factors contributing to the acoustic signals related to Parkinson’s disease. This section discusses the evaluation of algorithms developed on the PD dataset for early diagnosis of PD at each category level. The systems achieved better results after applying the t-SNE and PCA algorithms. This means that the existence of some features is negatively associated with the target feature and affects the efficiency of the system.

**Figure 5:**
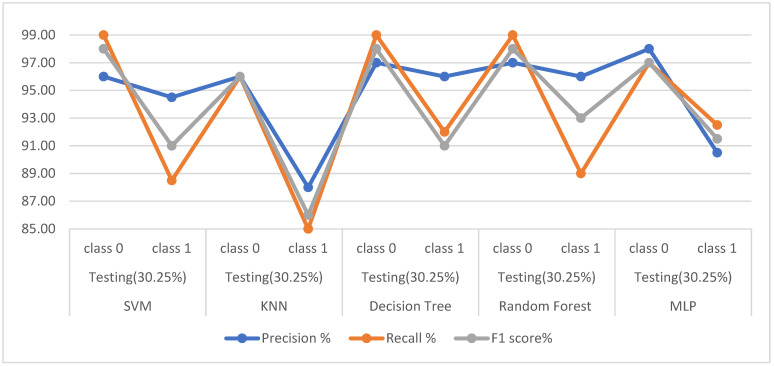
Display the performance of the classifiers for each class with t-SEN.

First, when the classifiers are fed the dataset produced by the t-SNE algorithm, Table 10 and Figure 5 describe the results of diagnosing the classes of the dataset, which are healthy (class 0) and PD (class 1). In the healthy class, it is noted that SVM, KNN, DT, RF, and MLP achieved the following results: for precision by 96%, 96%, 97%, 97%, and 98%, respectively; also for recall by 99%, 96%, 99%, 99%, and 97%, respectively; while the for F1-score 98%, 96%, 98%, 98%, and 97%, respectively. For the PD class, it is noted that SVM, KNN, DT, RF, and MLP achieved the following results: for precision 94%, 88%, 96%, 96%, and 90.50%, respectively; and for recall at 88.50%, 85%, 92%, 89%, and 92.50%, respectively; while the F1-score by 91%, 86%, 91%, 93%, and 91.50%, respectively.

Our findings suggest that acoustic signals can be used to detect Parkinson’s disease automatically and early. This could lead to earlier diagnosis and treatment, which could improve the quality of life for people with Parkinson’s disease.

The systems optimize the early diagnosis of PD by evaluating selected features and hyperparameter tuning of ML algorithms for diagnosing PD based on voice disorders. The study found that the proposed techniques were superior to existing studies, indicating the superiority of the proposed techniques. This contributes to the evaluation and advancement of existing knowledge in the field of Parkinson’s disease diagnosis.

**Figure 6:**
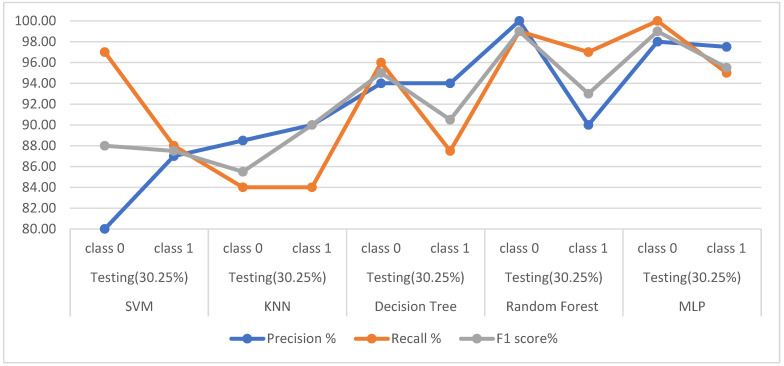
Display the performance of the classifiers for each class with PCA.

Second, when the classifiers are fed the dataset after dimensionality reduction by the PCA algorithm, Table 11 and Figure 6 describe the diagnostic results for each class. In the healthy class, it is noted that SVM, KNN, DT, RF, and MLP achieved the following results: for precision by 80%, 88.50%, 94%, 100%, and 98%, respectively; also for recall by 97%, 84%, 96%, 99%, and 100%, respectively; while the for F1-score 88%, 85.50%, 95%, 99%, and 99%, respectively. For the PD class, it is noted that SVM, KNN, DT, RF, and MLP achieved the following results: for precision 87%, 90%, 94%, 90%, and 97.50%, respectively; and for recall at 88%, 84%, 87.50%, 97%, and 95%, respectively; while the F1-score by 87.50%, 90%, 90.50%, 93%, and 95.50%, respectively.

**Figure 7:**
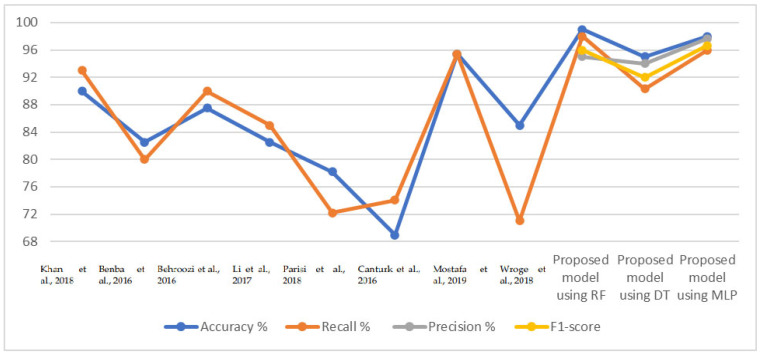
Display of a comparison of the performance of some of our classifiers with some previous studies [31,32,63,64,65,67,69,70].

Table 12 and Figure 7 present a comparison of the results of the proposed classification models with existing models discussed in the literature. Our proposed model provides better results over existing studies, whereas the previous studies achieved an accuracy score between 95.43% and 78.23%, while the proposed systems reached an accuracy of 98%, 96%, and 98% for the RF, DT, and MLP classifiers, respectively. The measure of recall (sensitivity) in the previous systems was between 95.4% and 71%, while those of the proposed systems were 98%, 93.66%, and 96% for RF, DT, and MLP classifiers, respectively.

**Table 12:** Comparison of performance of the proposed system with previous studies.

Previous Studies	Accuracy%	Recall%	Precision%	F1-Score
Khan et al. [63]	90	93	-	-
Benba et al. [64]	82.5	80	-	-
Behroozi et al. [65]	87.5	90	-	-
Li et al. [66]	82.5	85	-	-
Parisi et al. [67]	78.23	72.22	-	-
Cantürk and Karabiber [68]	68.94	74.03	-	-
Mostafa et al. [69]	95.43	95.4	-	-
Wroge et al. [70]	85	71		
Proposed model using RF	99	98	95	96
Proposed model using DT	95	90.33	94	92
Proposed model using MLP	98	96	97.66	96.66

Reliability refers to the consistency and stability of measurements or techniques, while validity refers to the accuracy and appropriateness of the measurements or techniques in assessing the intended construct or phenomenon. Fortunately, the information provided does explicitly mention the reliability and validity of the measures used in the study. The study reported the accuracy of the proposed techniques through the accuracy of the results assessed thanks to the reliability and validity of the measures used. The study stated that the proposed techniques were able to identify PD with an accuracy of up to 98%. Overall, the study provides some promising evidence that acoustic signals can be used to detect PD automatically and early.

The implications of the findings are significant for the field of Parkinson’s disease diagnosis and treatment. By leveraging machine-learning algorithms and analyzing acoustic signals, they have demonstrated the potential for automated and early detection of PD. This approach offers several advantages over traditional diagnosis methods, which often involve time-consuming physical and psychological tests and specialist examinations of the patient’s nervous system. By extracting a set of features from recordings of a person’s voice, we were able to train machine-learning models to distinguish between Parkinson’s cases and healthy individuals. This noninvasive approach has the potential to revolutionize the early detection of PD, enabling timely interventions and improved patient outcomes. The study also introduces two techniques, t-SNE and PCA, for the dimensionality reduction of the dataset. By reducing the number of features while retaining the most informative ones, these techniques help improve the efficiency and performance of the classification algorithms. The experimental results presented in the paper demonstrate the effectiveness of the proposed techniques. These findings have practical implications for healthcare professionals involved in PD diagnosis. The proposed techniques can be applied to develop automated systems that assist in the early screening and diagnosis of PD based on voice analysis. Such systems could potentially be integrated into routine clinical practice, enabling cost-effective and widespread screening for PD, particularly in populations where access to specialized neurological examinations may be limited. Furthermore, the study contributes to the existing knowledge by demonstrating the efficacy of specific machine-learning algorithms (RF and MLP) and dimensionality reduction techniques (t-SNE and PCA) in the context of PD diagnosis. This knowledge can inform future studies and inspire further study into the development of advanced diagnostic tools and methodologies for Parkinson’s disease.

Here are some potential limitations and biases to consider: the study’s methodology relies on analyzing voice recordings to diagnose Parkinson’s disease. It is essential to discuss the specifics of the data-collection process, including the recording equipment used, the recording environment, and any potential limitations or sources of error introduced during data acquisition. The RFE algorithm selected relevant features from the voice recordings. The criteria used for feature selection and the potential impact of excluding certain features. Biases may arise if certain features are overrepresented or if crucial features are unintentionally omitted.

## 6. Conclusions

PD is a disease caused by a lack of dopamine, which affects the elderly and disrupts their lives. This disease is difficult to diagnose because its symptoms are unclear and associated with other diseases. Extensive medical and scientific research has been conducted to diagnose PD early. ML techniques have contributed to early diagnosis by analysing a person’s voice disorders. The present study contributes knowledge useful for the early diagnosis of PD by providing a voice dataset comprising 22 features. These features appeared highly correlated, thereby making them unsuitable for high-level diagnosis. The features that contained outliers were removed. The RFE algorithm was applied to rank the features according to their importance; then, the dimensions were reduced by using two algorithms, t-SNE and PCA, to represent the data in a low-dimensional space. The SVM, KNN, DT, RF, and MLP classifiers were fed the resulting features by both t-SNE and PCA algorithms. All classifiers achieved superior results for diagnosing PD and normal cases. During the testing phase, RF with the t-SNE algorithm achieved an accuracy of 97%, precision of 96.50%, recall of 94%, and F1-score of 95%. While MLP with the PCA algorithm achieved an accuracy of 98%, precision of 97.66%, recall of 96%, and F1-score of 96.66%. We have shown that acoustic signals can be used to detect Parkinson’s disease automatically and early. This is a significant finding, as it could lead to earlier diagnosis and treatment, which could improve the quality of life for people with Parkinson’s disease. Our findings contribute to existing knowledge by providing a new method for detecting Parkinson’s disease.

## Figures and Tables

**Table 1:** PD Dataset with interpretation of voice measures.

No	Voice Measure	Description	Dtype
1	MDVP: Fo (Hz)	Average_vocal_fundamental_frequency	float64
2	MDVP: Fhi (Hz)	Maximum_vocal_fundamental_frequency	float64
3	MDVP: Flo (Hz)	Minimum_vocal_fundamental_frequency	float64
4	MDVP: Jitter (%)	Several_measures_of_variation-in	float64
5	MDVP: Jitter (Abs)	Fundamental_frequency	float64
6	MDVP: RAP		float64
7	MDVP: PPQ		float64
8	Jitter: DDP		float64
9	MDVP: Shimmer	Several_measures_of_variation_in_amplitude	float64
10	MDVP: Shimmer (dB)		float64
11	Shimmer: APQ3		float64
12	Shimmer: APQ5		float64
13	MDVP: APQ		float64
14	Shimmer: DDA		float64
15	NHR	Two measures_of_ratio_of_noise_to_tonal	float64
16	HNR	Components_in_the_voice	float64
17	Status	Health_status_of-the_subject:1 Parkinson’s,0-healthy	object
18	RPDE	Two_nonlinear_dynamical_complexity	float64
19	DFA	Signal_fractal_scaling_exponent	float64
20	Spread1	Three_nonlinear_measures_of_fundamental	float64
21	Spread2	Frequency_variation	float64
22	D2	Measures	float64
23	PPE		float64

**Table 2:** Value of skewness for the dataset.

Feature No.	Voice Measure	Skewness Value
1	MDVP: Fo (Hz)	0.5872
2	MDVP: Fhi (Hz)	2.5225
3	MDVP: Flo (Hz)	1.208
4	MDVP: Jitter (%)	3.0612
5	MDVP: Jitter (Abs)	2.6287
6	MDVP: RAP	3.3348
7	MDVP: PPQ	3.0502
8	Jitter: DDP	3.3361
9	MDVP: Shimmer	1.6536
10	MDVP: Shimmer (dB)	1.984
11	Shimmer: APQ3	1.5684
12	Shimmer: APQ5	1.7848
13	MDVP: APQ	2.5979
14	Shimmer: DDA	1.5684
15	NHR	4.1882
16	HNR	0.5104
17	RPDE	−0.1423
18	DFA	−0.033
19	spread1	0.4288
20	spread2	0.1433
21	D2	0.4271
22	PPE	0.7913

**Table 3:** Value of CV for the dataset.

Feature No.	Voice Measure	Coefficient of Variation	
		Healthy	PD Patients
1	MDVP: Fo (Hz)	28.98302	22.2812234
2	MDVP: Fhi (Hz)	43.25187	46.87884421
3	MDVP: Flo (Hz)	40.46427	30.19298691
4	MDVP: Jitter (%)	53.14539	74.96921231
5	MDVP: Jitter (Abs)	64.02608	72.61445527
6	MDVP: RAP	55.38599	86.25906336
7	MDVP: PPQ	45.87769	76.8525638
8	Jitter: DDP	55.39028	86.25436548
9	MDVP: Shimmer	31.47526	59.33205227
10	MDVP: Shimmer (dB)	35.48279	64.69356531
11	Shimmer: APQ3	36.36572	61.08510866
12	Shimmer: APQ5	30.74724	63.33464804
13	MDVP: APQ	28.74997	65.44260656
14	Shimmer: DDA	36.366	61.08457075
15	NHR	166.2361	152.1592919
16	HNR	13.91699	20.68815053
17	RPDE	20.8335	19.59196532
18	DFA	7.380315	7.552488454
19	spread1	−9.50964	−18.2020606
20	spread2	39.29221	31.35796616
21	D2	14.40103	15.298566
22	PPE	36.43383	36.03981785

**Table 4:** SMOTE method for balancing the dataset.

Phase	Training 69.75%		Testing 30.25%	
Classes	Healthy	Parkinson	Healthy	Parkinson
Before OverSampling	34	103	14	44
After OverSampling	103	103	14	44

**Table 5:** Standardizing Continuous Values for a PD Dataset.

No	MDVP: Fo (Hz)	MDVP: Fhi (Hz)	MDVP: Flo (Hz)	MDVP: Jitter	Spread1	Spread2	D2
0	−0.8285	−0.4355	−0.9404	0.8076	0.9733	0.5550	−0.1360
1	−0.7709	−0.5781	−0.0551	1.4311	1.7109	1.4101	0.3872
2	−0.9075	−0.8670	−0.1067	1.7090	1.3430	1.1080	−0.0208
3	−0.9077	−0.7557	−0.1110	1.5294	1.6686	1.3923	0.1578
4	−0.9235	−0.6912	−0.1273	2.5019	2.0382	0.1594	−0.0493

**Table 6:** Contribution of each feature to the diagnosis of Parkinson’s.

Features	Priority%
MDVP: Fo (Hz)	23
MDVP: Fhi (Hz)	11
MDVP: Flo (Hz)	14.5
MDVP: Jitter (%)	4.1
MDVP: Shimmer	6.1
NHR	6.4
HNR	1.3
RPDE	0.2
DFA	0.4
spread1	27.6
spread2	4.9
D2	0.5

**Table 7:** System implementation environment.

Resource	Details
CPU	Core i5 Gen6
RAM 8 GB	12 GB
GPU	4 GB
Software	Python

**Table 8:** Results of Parkinson’s diagnosis using classifiers with the t-SNE method.

Classifiers	SVM		KNN		Decision Tree		Random Forest		MLP	
Criteria	Training 69.75%	Testing 30.25%	Training 69.75%	Testing 30.25%	Training 69.75%	Testing 30.25%	Training 69.75%	Testing 30.25%	Training 69.75%	Testing 30.25%
Accuracy%	95.72	96.00	96.00	93.00	97.00	96.00	98.00	97.00	97.00	96.00
Precision%	92.80	95.00	94.66	90.66	96.33	93.33	97.50	96.50	95.82	93.00
Recall%	92.66	92.00	91.66	88.66	94.33	93.66	99.00	94.00	94.22	94.00
F1 score	93.57	93.33	93.00	89.33	95.33	93.33	98.50	95.00	95.01	93.49

**Table 9:** Results of Parkinson’s diagnosis using classifiers with the PCA method.

Classifiers	SVM		KNN		Decision Tree		Random Forest		MLP	
Criteria	Training 69.75%	Testing 30.25%	Training 69.75%	Testing 30.25%	Training 69.75%	Testing 30.25%	Training 69.75%	Testing 30.25%	Training 69.75%	Testing 30.25%
Accuracy%	94.00	93.00	94.00	93.00	96.00	95.00	99.00	99.00	98.43	98.00
Precision%	90.00	84.70	89.30	91.00	91.33	94.00	95.52	95.00	98.00	97.66
Recall%	91.66	91.20	89.00	84.00	93.66	90.33	99.10	98.00	97.66	96.00
F1 score	90.66	87.70	89.03	87.00	92.66	92.00	97.28	96.00	98.00	96.66

**Table 10:** Results of each class Parkinson’s diagnosis with the t-SNE method.

Classifiers	SVM		KNN		Decision Tree		Random Forest		MLP	
Phase	Testing (30.25%)		Testing (30.25%)		Testing (30.25%)		Testing (30.25%)		Testing (30.25%)	
Class	Class 0	Class 1	Class 0	Class 1	Class 0	Class 1	Class 0	Class 1	Class 0	Class 1
Precision%	96.00	94.50	96.00	88.00	97.00	96.00	97.00	96.00	98.00	90.50
Recall%	99.00	88.50	96.00	85.00	99.00	92.00	99.00	89.00	97.00	92.50
F1 score%	98.00	91.00	96.00	86.00	98.00	91.00	98.00	93.00	97.00	91.50

**Table 11:** Results of each class Parkinson’s diagnosis with the PCA method.

Classifiers	SVM		KNN		Decision Tree		Random Forest		MLP	
Phase	Testing (30.25%)		Testing (30.25%)		Testing (30.25%)		Testing (30.25%)		Testing (30.25%)	
Class	Class 0	Class 1	Class 0	Class 1	Class 0	Class 1	Class 0	Class 1	Class 0	Class 1
Precision%	80.00	87.00	88.50	90.00	94.00	94.00	100.00	90.00	98.00	97.50
Recall%	97.00	88.00	84.00	84.00	96.00	87.50	99.00	97.00	100.00	95.00
F1-score%	88.00	87.50	85.50	90.00	95.00	90.50	99.00	93.00	99.00	95.50

## Data Availability

The data applied to this study to assess systems performance were obtained from the UCI Machine Learning Repository: Parkinsons dataset, which is publicly available at: https://archive.ics.uci.edu/ml/datasets/parkinsons (accessed on 23 February 2022).

## References

[1] Wang Z.L., Yuan L., Li W., Li J.Y. (2022). Ferroptosis in Parkinson’s disease: Glia–neuron crosstalk. Trends Mol. Med..

[2] Yousefvand S., Hamidi F. (2020). Role of Lateral Hypothalamus Area in the Central Regulation of Feeding. Int. J. Pept. Res. Ther..

[3] Panda A., Bhuyan P. (2022). Machine Learning-Based Framework for Early Detection of Distinguishing Different Stages of Parkinson’s Disease. Spec. Ugdym..

[4] Behl T., Makkar R., Sehgal A., Sharma N., Singh S., Albratty M., Bungau S.G. (2022). Insights into the Explicit Protective Activity of Herbals in Management of Neurodegenerative and Cerebrovascular Disorders. Molecules.

[5] Alfonsetti M., Castelli V., d’Angelo M. (2022). Are We What We Eat? Impact of Diet on the Gut–Brain Axis in Parkinson’s Disease. Nutrients.

[6] Wang L., Gao Z., Chen G., Geng D., Gao D. (2023). Low Levels of Adenosine and GDNF Are Potential Risk Factors for Parkinson’s Disease with Sleep Disorders. Brain Sci..

[7] Mata-Marín D., Pineda-Pardo J.A., Molina J.A., Vela L., Alonso-Frech F., Obeso I. (2021). Aberrant salient and corticolimbic connectivity in hypersexual Parkinson’s disease. Brain Connect..

[8] Ngo Q.C., Motin M.A., Pah N.D., Drotár P., Kempster P., Kumar D. (2022). Computerized analysis of speech and voice for Parkinson’s disease: A systematic review. Comput. Methods Programs Biomed..

[9] Barukab O., Ahmad A., Khan T., Thayyil Kunhumuhammed M.R. (2022). Analysis of Parkinson’s Disease Using an Imbalanced-Speech Dataset by Employing Decision Tree Ensemble Methods. Diagnostics.

[10] Domingos J., Dean J., Fernandes J.B., Godinho C. (2022). Professionals’ Self-Reported Difficulties towards Integrating Dual Task Training in Care for People with Parkinson’s Disease. Int. J. Environ. Res. Public Health.

[11] Ali L., Chakraborty C., He Z., Cao W., Imrana Y., Rodrigues J.J. (2022). A novel sample and feature dependent ensemble approach for Parkinson’s disease detection. Neural Comput. Appl..

[12] Liu C., Fan Z., He D., Chen H., Zhang S., Guo S., Zheng B., Cen H., Zhao Y., Liu H. (2022). Designer Functional Nanomedicine for Myocardial Repair by Regulating the Inflammatory Microenvironment. Pharmaceutics.

[13] Ufer C., Blank H. (2023). Multivariate analysis of brain activity patterns as a tool to understand predictive processes in speech perception. Lang. Cogn. Neurosci..

[14] Khaskhoussy R., Ayed Y.B. An I-vector-based approach for discriminating between patients with Parkinson’s disease and healthy people. Proceedings of the Fourteenth International Conference on Machine Vision.

[15] Saravanan S., Ramkumar K., Adalarasu K., Sivanandam V., Kumar S.R., Stalin S., Amirtharajan R. (2022). A systematic review of artificial intelligence (AI) based approaches for the diagnosis of Parkinson’s disease. Arch. Comput. Methods Eng..

[16] Maskeliūnas R., Damaševičius R., Kulikajevas A., Padervinskis E., Pribuišis K., Uloza V. (2022). A Hybrid U-Lossian Deep Learning Network for Screening and Evaluating Parkinson’s Disease. Appl. Sci..

[17] Ahmadi A., Bazregarzadeh H., Kazemi K. (2021). Automated detection of driver fatigue from electroencephalography through wavelet-based connectivity. Biocybern. Biomed. Eng..

[18] Sánchez-Hernández S.E., Salido-Ruiz R.A., Torres-Ramos S., Román-Godínez I. (2022). Evaluation of Feature Selection Methods for Classification of Epileptic Seizure EEG Signals. Sensors.

[19] Davoudi S., Ahmadi A., Daliri M.R. (2021). Frequency—Amplitude coupling: A new approach for decoding of attended features in covert visual attention task. Neural Comput. Appl..

[20] Anuragi A., Sisodia D.S., Pachori R.B. (2022). EEG-based cross-subject emotion recognition using Fourier-Bessel series expansion based empirical wavelet transform and NCA feature selection method. Inf. Sci..

[21] Sakalle A., Tomar P., Bhardwaj H., Iqbal A., Sakalle M., Bhardwaj A., Ibrahim W. (2022). Genetic programming-based feature selection for emotion classification using EEG signal. J. Healthc. Eng..

[22] Loh H.W., Ooi C.P., Palmer E., Barua P.D., Dogan S., Tuncer T., Baygin M., Acharya U.R. (2021). GaborPDNet: Gabor Transformation and Deep Neural Network for Parkinson’s Disease Detection Using EEG Signals. Electronics.

[23] Aljalal M., Aldosari S.A., AlSharabi K., Abdurraqeeb A.M., Alturki F.A. (2022). Parkinson’s Disease Detection from Resting-State EEG Signals Using Common Spatial Pattern, Entropy, and Machine Learning Techniques. Diagnostics.

[24] Borzì L., Mazzetta I., Zampogna A., Suppa A., Olmo G., Irrera F. (2021). Prediction of Freezing of Gait in Parkinson’s Disease Using Wearables and Machine Learning. Sensors.

[25] Chintalapudi N., Battineni G., Hossain M.A., Amenta F. (2022). Cascaded Deep Learning Frameworks in Contribution to the Detection of Parkinson’s Disease. Bioengineering.

[26] Rana A., Dumka A., Singh R., Rashid M., Ahmad N., Panda M.K. (2022). An Efficient Machine Learning Approach for Diagnosing Parkinson’s Disease by Utilizing Voice Features. Electronics.

[27] Khachnaoui H., Khlifa N., Mabrouk R. (2022). Machine Learning for Early Parkinson’s Disease Identification within SWEDD Group Using Clinical and DaTSCAN SPECT Imaging Features. J. Imaging.

[28] Pramanik M., Pradhan R., Nandy P., Bhoi A.K., Barsocchi P. (2021). Machine Learning Methods with Decision Forests for Parkinson’s Detection. Appl. Sci..

[29] Sakar B.E., Isenkul M.E., Sakar C.O., Sertbas A., Gurgen F., Delil S., Kursun O. (2013). Collection and analysis of a Parkinson speech dataset with multiple types of sound recordings. IEEE J. Biomed. Health Inform..

[30] Little M., McSharry P., Hunter E., Spielman J., Ramig L. (2008). Suitability of dysphonia measurements for telemonitoring of Parkinson’s disease. Nat. Preced..

[31] Cantürk İ., Karabiber F. (2016). A machine learning system for the diagnosis of Parkinson’s disease from speech signals and its application to multiple speech signal types. Arab. J. Sci. Eng..

[32] Li Y., Zhang C., Jia Y., Wang P., Zhang X., Xie T. Simultaneous learning of speech feature and segment for classification of Parkinson disease. Proceedings of the 2017 IEEE 19th International Conference on e-Health Networking, Applications and Services.

[33] Benba A., Jilbab A., Hammouch A. (2017). Using human factor cepstral coefficient on multiple types of voice recordings for detecting patients with Parkinson’s disease. IRBM.

[34] Almeida J.S., Rebouças Filho P.P., Carneiro T., Wei W., Damaševičius R., Maskeliūnas R., de Albuquerque V.H.C. (2019). Detecting Parkinson’s disease with sustained phonation and speech signals using machine learning techniques. Pattern Recognit. Lett..

[35] Das R. (2010). A comparison of multiple classification methods for diagnosis of Parkinson disease. Expert Syst. Appl..

[36] Yuvaraj R., Murugappan M., Ibrahim N.M., Sundaraj K., Omar M.I., Mohamad K., Palaniappan R. (2014). Detection of emotions in Parkinson’s disease using higher order spectral features from brain’s electrical activity. Biomed. Signal Process. Control.

[37] Direito B., Teixeira C.A., Sales F., Castelo-Branco M., Dourado A. (2017). A realistic seizure prediction study based on multiclass SVM. Int. J. Neural Syst..

[38] Acharya U.R., Sree S.V., Suri J.S. (2011). Automatic detection of epileptic EEG signals using higher order cumulant features. Int. J. Neural Syst..

[39] Yuvaraj R., Rajendra Acharya U., Hagiwara Y. (2018). A novel Parkinson’s Disease Diagnosis Index using higher-order spectra features in EEG signals. Neural Comput. Appl..

[40] Sivaranjini S., Sujatha C.M. (2020). Deep learning based diagnosis of Parkinson’s disease using convolutional neural network. Multimed. Tools Appl..

[41] Ali L., Zhu C., Zhou M., Liu Y. (2019). Early diagnosis of Parkinson’s disease from multiple voice recordings by simultaneous sample and feature selection. Expert Syst. Appl..

[42] Senturk Z.K. (2020). Early diagnosis of Parkinson’s disease using machine learning algorithms. Med. Hypotheses.

[43] Gupta D., Sundaram S., Khanna A., Hassanien A.E., De Albuquerque V.H.C. (2018). Improved diagnosis of Parkinson’s disease using optimized crow search algorithm. Comput. Electr. Eng..

[44] UCI Machine Learning Repository: Parkinsons Data Set. https://archive.ics.uci.edu/ml/datasets/parkinsons.

[45] Khalid A., Senan E.M., Al-Wagih K., Ali Al-Azzam M.M., Alkhraisha Z.M. (2023). Hybrid Techniques of X-ray Analysis to Predict Knee Osteoarthritis Grades Based on Fusion Features of CNN and Handcrafted. Diagnostics.

[46] Senan E.M., Jadhav M.E., Rassem T.H., Aljaloud A.S., Mohammed B.A., Al-Mekhlafi Z.G. (2022). Early diagnosis of brain tumour mri images using hybrid techniques between deep and machine learning. Comput. Math. Methods Med..

[47] Wang J., Zhou Z., Li Z., Du S. (2022). A Novel Fault Detection Scheme Based on Mutual k-Nearest Neighbor Method: Application on the Industrial Processes with Outliers. Processes.

[48] Puggard W., Niwitpong S.A., Niwitpong S. (2022). Confidence intervals for common coefficient of variation of several Birnbaum–Saunders distributions. Symmetry.

[49] GhoshRoy D., Alvi P.A., Santosh K. (2023). Explainable AI to Predict Male Fertility Using Extreme Gradient Boosting Algorithm with SMOTE. Electronics.

[50] Tesfahun A., Bhaskari D.L. Intrusion detection using random forests classifier with SMOTE and feature reduction. Proceedings of the 2013 International Conference on Cloud & Ubiquitous Computing & Emerging Technologies.

[51] Wotzka D., Sikorski W., Szymczak C. (2022). Investigating the Capability of PD-Type Recognition Based on UHF Signals Recorded with Different Antennas Using Supervised Machine Learning. Energies.

[52] Chang K.-H., Wang C.-H., Hsu B.-G., Tsai J.-P. (2022). Serum Osteopontin Level Is Positively Associated with Aortic Stiffness in Patients with Peritoneal Dialysis. Life.

[53] Fati S.M., Senan E.M., Azar A.T. (2022). Hybrid and Deep Learning Approach for Early Diagnosis of Lower Gastrointestinal Diseases. Sensors.

[54] Ullah B., Kamran M., Rui Y. (2022). Predictive Modeling of Short-Term Rockburst for the Stability of Subsurface Structures Using Machine Learning Approaches: T-SNE, K-Means Clustering and XGBoost. Mathematics.

[55] Dweekat O.Y., Lam S.S. (2022). Cervical Cancer Diagnosis Using an Integrated System of Principal Component Analysis, Genetic Algorithm, and Multilayer Perceptron. Healthcare.

[56] Tsai F., Lin E.K., Yoshino K. (2007). Spectrally segmented principal component analysis of hyperspectral imagery for mapping invasive plant species. Int. J. Remote Sens..

[57] Senan E.M., Abunadi I., Jadhav M.E., Fati S.M. (2021). Score and Correlation Coefficient-Based Feature Selection for Predicting Heart Failure Diagnosis by Using Machine Learning Algorithms. Comput. Math. Methods Med..

[58] Senan E.M., Jadhav M.E., Kadam A. Classification of PH2 images for early detection of skin diseases. Proceedings of the 2021 6th International Conference for Convergence in Technology.

[59] Senan E.M., Jadhav M.E. (2022). Diagnosis of dermoscopy images for the detection of skin lesions using SVM and KNN. Proceedings of the Third International Conference on Sustainable Computing.

[60] Steigmann L., Di Gianfilippo R., Steigmann M., Wang H.-L. (2022). Classification Based on Extraction Socket Buccal Bone Morphology and Related Treatment Decision Tree. Materials.

[61] Ahmed I.A., Senan E.M., Shatnawi H.S.A., Alkhraisha Z.M., Al-Azzam M.M.A. (2023). Hybrid Techniques for the Diagnosis of Acute Lymphoblastic Leukemia Based on Fusion of CNN Features. Diagnostics.

[62] Mohammed B.A., Senan E.M., Rassem T.H., Makbol N.M., Alanazi A.A., Al-Mekhlafi Z.G., Almurayziq T.S., Ghaleb F.A. (2021). Multi-Method Analysis of Medical Records and MRI Images for Early Diagnosis of Dementia and Alzheimer’s Disease Based on Deep Learning and Hybrid Methods. Electronics.

[63] Khan M.M., Mendes A., Chalup S.K. (2018). Evolutionary Wavelet Neural Network ensembles for breast cancer and Parkinson’s disease prediction. PLoS ONE.

[64] Benba A., Jilbab A., Hammouch A. (2016). Analysis of multiple types of voice recordings in cepstral domain using MFCC for discriminating between patients with Parkinson’s disease and healthy people. Int. J. Speech Technol..

[65] Behroozi M., Sami A. (2016). A multiple-classifier framework for Parkinson’s disease detection based on various vocal tests. Int. J. Telemed. Appl..

[66] Khalid A., Senan E.M., Al-Wagih K., Al-Azzam M.M.A., Alkhraisha Z.M. (2023). Automatic Analysis of MRI Images for Early Prediction of Alzheimer’s Disease Stages Based on Hybrid Features of CNN and Handcrafted Features. Diagnostics.

[67] Parisi L., RaviChandran N., Manaog M.L. (2018). Feature-driven machine learning to improve early diagnosis of Parkinson’s disease. Expert Syst. Appl..

[68] Al-Jabbar M., Alshahrani M., Senan E.M., Ahmed I.A. (2023). Multi-Method Diagnosis of Histopathological Images for Early Detection of Breast Cancer Based on Hybrid and Deep Learning. Mathematics.

[69] Mostafa S.A., Mustapha A., Mohammed M.A., Hamed R.I., Arunkumar N., Abd Ghani M.K., Khaleefah S.H. (2019). Examining multiple feature evaluation and classification methods for improving the diagnosis of Parkinson’s disease. Cogn. Syst. Res..

[70] Wroge T.J., Özkanca Y., Demiroglu C., Si D., Atkins D.C., Ghomi R.H. Parkinson’s disease diagnosis using machine learning and voice. Proceedings of the 2018 IEEE Signal Processing in Medicine and Biology Symposium, IEEE.

